# Using deep learning to classify developmental differences in reaching and placing movements in children with and without autism spectrum disorder

**DOI:** 10.1038/s41598-024-81652-z

**Published:** 2024-12-05

**Authors:** Wan-Chun Su, John Mutersbaugh, Wei-Lun Huang, Anjana Bhat, Amir Gandjbakhche

**Affiliations:** 1 Eunice Kennedy Shriver National Institute of Child Health and Human Development (NICHD), National Institutes of Health, Building 49, Room 5A82, 49 Convent Drive, Bethesda, MD 20892-4480 USA; 2 School of Kinesiology, Louisiana State University, Baton Rouge, LA USA; 3 Department of Computer Science, Johns Hopkins University, Baltimore, MD USA; 4 Department of Physical Therapy, University of Delaware, Newark, DE USA; 5 Biomechanics & Movement Science Program, University of Delaware, Newark, DE USA; 6 Department of Psychological & Brain Sciences, University of Delaware, Newark, DE USA

**Keywords:** Motor control, Sensorimotor processing, Neuroscience, Signs and symptoms

## Abstract

Autism Spectrum Disorder (ASD) is among the most prevalent neurodevelopmental disorders, yet the current diagnostic procedures rely on behavioral analyses and interviews, without objective screening methods to support the diagnostic process. This study seeks to address this gap by integrating upper limb kinematics and deep learning methods to identify potential biomarkers that could be validated in younger age groups in the future to enhance the identification of ASD. Forty-one school-age children, with and without an ASD diagnosis (mean age ± SE: TD group: 10.3 ± 0.8, 8 males and 7 females; ASD group: 10.3 ± 0.5, 21 males and 5 females), participated in the study. A single Inertial Measurement Unit (IMU) was affixed to the child’s wrist as they engaged in a continuous reaching and placing task. Deep learning techniques were employed to classify children with and without ASD. Our findings suggest differential movement kinematics in school-age children compared to healthy adults. Compared to TD children, children with ASD exhibited poor feedforward/feedback control of arm movements as seen by greater number of movement units, more movement overshooting, and prolonged time to peak velocity/acceleration. Unique movement strategies such as greater velocity and acceleration were also seen in the ASD group. More importantly, using Multilayer Perceptron (MLP) model, we demonstrated an accuracy of ~ 78.1% in classifying children with and without ASD. These findings underscore the potential use of studying upper limb movement kinematics during goal-directed arm movements and deep learning methods as valuable tools for classifying and, consequently, aiding in the diagnosis and early identification of ASD upon further validation of their specificity among younger children.

## Introduction

Autism Spectrum Disorder (ASD) ranks among the most prevalent neurodevelopmental disorders in the U.S., affecting approximately 1 in 36 children^1^. As defined by the Diagnostic and Statistical Manual of Mental Disorders, 5th Edition, children with ASD exhibit distinct characteristics, including social communication difficulties and the presence of repetitive behaviors^2^. However, due to the disorder’s high heterogeneity, diagnosing ASD is still very challenging^3^. The current diagnostic process for ASD relies on behavioral analyses and interviews, without well-established objective biomarkers^3^. Additionally, the core diagnostic features, such as social communication difficulties, often do not become apparent until later stages of development, limiting their utility in early ASD identification^4^. While not constituting the primary diagnostic criteria, motor impairments are highly prevalent and significantly related to other social communication difficulties of children with ASD^5–7^. More importantly, many motor signs, such as atypical reach-to-grasp movements, manifest early in life and hold promise as diagnostic biomarkers^4,8^. Given the potential value of studying objective motor measures, our current study explored the developmental differences in arm movement kinematics during goal-directed reaching and the application of deep learning algorithms for the classification of Typically Developing (TD) children and children with ASD. Our findings provide deeper insights into motor development and hold significant clinical potential for early ASD identification upon further validation of the specificity among younger children.

During early development, early spontaneous arm movements change into more refined, object-oriented actions, with object contact and grasping emerging between 4 to 6 months of age^9–11^. Subsequent to the onset of grasping, reaching movements undergo further refinement, adopting a smoother and more fluent trajectory through feedback corrections to initial reaches^12^. A longitudinal analysis of hand and joint kinematics during spontaneous and purposeful reaching from 8 to 20 weeks indicated gradual increase in movement length and more purposeful reaching in presence of a toy^9–11^. In addition, early on infants controlled their shoulder motions and later close to reach onset controlled both shoulder and elbow motions to make toy contact^13,14^. This refinement in reaching movements will continue to persist into childhood. A study targeting school-age children found higher peak velocity and straighter movement trajectories in children aged 11–15 years old compared to 5 to 7 years old children when reaching toward different directions^15^. In the current study, our focus extends to typically developing school-age children, as well as those diagnosed with ASD. We aim to compare their reaching and placing movements with those of healthy adults to examine differences in reaching trajectories between the two groups.

Although not included in the primary diagnostic criteria which mainly include social communication difficulties and presence of repetitive behaviors, children with ASD exhibit challenges in both gross and fine motor performance, including motor incoordination, deficient posture control, as well as poor upper limb motor control/dexterity^16–19^. Recent insights gleaned from parent screening questionnaires in the SPARK database reveal that over 85% of children with ASD are at risk for motor difficulties, underscoring the critical importance of recognizing and addressing these motor issues within this population^20^. Documentation of motor challenges in children with ASD also goes beyond screening tools and behavioral assessments; and extends to video coding and quantitative measures such as electromyographic (EMG) activity and movement kinematics^21–25^. Children with ASD exhibit poor feedforward and feedback control of their arm movements^21–25^. Studies on feeding and reaching movements suggest that children with ASD exhibit insufficient anticipatory movement control^21,22^. For example, Brisson et al.^21^ analyzed approximately 97 home videos and found that infants later diagnosed with ASD opened their mouths less frequently in anticipation of a spoon approaching during caregiver feeding. Similarly, Cattaneo et al. (2007) used EMG and observed delayed activation of the mylohyoid muscle (responsible for mouth opening) in children with ASD during grasp-to-eat movements compared to their TD peers, indicating impaired feedforward (motor planning) mechanisms^22^. In addition to impaired feedforward control, kinematic analyses of goal-directed arm and finger movements also indicate that children with ASD exhibit insufficient feedback/online control^23–25^. Yang et al.^24^ revealed that children with ASD, when reaching to grasp a cylinder-shaped target, displayed prolonged movement times, increased jerks, and a greater number of movement units compared to their TD peers. Similarly, Chua et al. (2022) used smart tablet games and found that children with ASD exhibited longer movement times, longer time to peak velocity, lower peak velocity, and a greater number of movement units after reaching peak velocity compared to TD children^23^. In a more complex motor task involving ball interceptions, children with ASD exhibited larger movement amplitudes, greater velocity, and more movement units compared to TD children^25^. Collectively, these findings suggest that children with ASD have impaired motor feedforward/feedback control during arm movements. In the current study, we used a child-friendly, lightweight motion-tracking system affixed to the children’s wrists to capture their upper movement kinematics during a reaching and placing task. Moreover, we extend the task from single to continuous, repetitive reaching and placing actions, making it a prolonged goal-directed action to detect ASD-related differences.

Motor difficulties observed in children with ASD have a profound impact on their daily living skills and are linked to various developmental domains^5–7^. Motor skills are known to serve as the foundation for the emergence of other social and cognitive skills during development^26,27^. For instance, locomotor skills facilitate the exploration of one’s surroundings, while manual dexterity fosters object manipulation and creates opportunities for joint attention and social interactions^26–29^. Recognizing the important role of motor skills in broader development, a comprehensive analysis of the SPARK database revealed that motor performance in children with ASD predicts developmental outcomes in social communication, repetitive behavior, language, and functional domains^6^. More importantly, motor signs in children with ASD often precede the emergence of social communication symptoms, making them an ideal biomarker for early identification^4^. Even in infancy, children later diagnosed with ASD exhibit less variability in general movements compared to their non-diagnosed counterparts^30^. In the current study, we seek to understand the relationships between the potential movement biomarkers and the adaptive functioning of children with and without ASD and explore the use of these biomarkers in classifying children with and without ASD.

Given the rapid growth of artificial intelligence techniques, a growing number of studies have turned to machine learning or deep learning methodologies for identifying children with ASD^31,32^. In a notable application, Heinsfeld et al.^31^ employed neuroimaging data from the Autism Brain Imaging Data Exchange, achieving a 78% accuracy in distinguishing children with ASD from healthy controls, with anterior–posterior connectivity identified as a significant contributor to this differentiation. On the other hand, Cila et al.^32^ attained an impressive approximately 90% accuracy in classifying children with and without ASD based on eye tracking data. To our knowledge, no deep learning study has utilized upper limb movement kinematics to classify children with and without ASD. Therefore, in the current study, we explore the movement kinematics of children with and without ASD during a reach-to-grasp task, investigating the relationships between these movement patterns with the children’s adaptive functioning. Additionally, we employ deep learning models to classify children with and without ASD based on their movement kinematics. To understand the general developmental trajectory of movement kinematics during goal-directed reach-and-place actions, and to determine where atypical movement kinematics in children with ASD may fall, we conducted statistical analyses comparing the movement kinematics of TD children and adults. Additionally, including typically developing participants in the study helps identify normative developmental trajectories and provides a reference to understanding atypical movement kinematics of autistic children. The outcomes of this study are poised to offer important insights into potential movement biomarkers for children with ASD, holding promise for their applications in the early identification of ASD in children upon further validation of their specificity among younger children.

## Methods

### Participants

Forty-one school-age children, with and without an ASD diagnosis, participated in the study (mean age ± SE: TD group: 10.3 ± 0.8, 8 males and 7 females; ASD group: 10.3 ± 0.5, 21 males and 5 females, Table 1). There were no significant differences in age, sex, race, and ethnicity between the two groups (Table 1). Recruitment of participants was conducted through online postings, phone calls, and distribution of fliers in local schools, community centers, ASD advocacy groups, and through the Simons Powering Autism Research (SPARK) participant research match service (https://www.sfari.org/resource/spark/). To ensure eligibility and gather essential demographic information such as age, sex, race, and ethnicity, we conducted screening interviews with parents via phone calls. For the TD group, children between 6 to 17 years were included and were excluded if they had any neurological or developmental diagnoses/delays, a history of preterm birth, significant birth complications, or a family history of ASD. In contrast, children in the ASD group were included if they met the following criteria: (1) their ages ranged from 6 to 17 years, (2) had a professionally confirmed ASD diagnosis, supported by either a school record (e.g., Individualized Education Plan) or medical/neuropsychological records from a qualified psychiatrist or clinical psychologist (using the Autism Diagnostic Observation Schedule (ADOS) or Autism Diagnostic Interview-Revised (ADI-R)^33,34^. On the other hand, children with ASD were excluded if they were unable to follow one-step instructions (e.g., “please pick up the blocks”), or if they exhibited significant sensory and behavioral challenges that hindered their ability to wear the fNIRS cap or complete the reaching-and-placing task. Additionally, parents were requested to complete the Vineland Adaptive Behavioral Scales- 2nd edition (VABS) and Social Responsive Scale (SRS) to assess their child’s adaptive functioning and social responsiveness (Table 1)^35,36^. Children with ASD were consistently found to have poor adaptive functions (indicated by lower VABS scores) and social responsiveness (indicated by higher SRS T score) compared to the TD group (*p*s < 0.05). Specifically, among the 15 TD children, 11 had adequate adaptive functioning, 2 were classified as moderately low, and 2 as moderately high or high. Among the children with ASD, 6 were classified as having adequate adaptive functioning, 12 as moderately low, and 8 as low. All study procedures were carried out in accordance with the Declaration of Helsinki. All inform consent and assent forms as well as all study procedures were approved by the University of Delaware Institutional Review Board (UD IRB). Before participating in the study, all children and their parents signed the inform consent or assent forms approved by the University of Delaware Institutional Review Board. Written parental and experimenter permission/consent to use their pictures for this publication has also been taken.

**Table 1:** Demographic information for participated children.

Characteristics	TD children (n = 15) Mean ± SE	ASD children (n = 26) Mean ± SE
Age	10.3 ± 0.8	10.3 ± 0.5
Sex	8M, 7F	21M, 5F
Race	9C, 1A, 1AA, 1AAA, 3AC	17C, 3A, 3AA, 1AAA, 1AC, 1CPI
Ethnicity	1H, 14NH	4H, 22NH
VABS-II (SS)	101.9 ± 4.5	76.8 ± 2.5*
Communication (SS)	103.4 ± 4.0	83.2 ± 3.0*
Daily living (SS)	101.4 ± 5.1	80.5 ± 2.7*
Socialization (SS)	100.2 ± 6.1	72.8 ± 2.9*
SRS (T scores)	57.2 ± 2.1	73.7 ± 1.7*

VABS-II = Vineland Adaptive Behavior Scale—2nd Edition; SS = Standard Score; SRS = Social Responsiveness Scale; M = Male; F = Female; C = Caucasian; A = Asian; AA = African American; AAA = Asian-African American; AC = Asian-Caucasian; CPI = Caucasian-Pacific Islander; H = Hispanic; NH = non-Hispanic. * Significant group differences (*p* < 0.05).

### Experimental procedure

**Fig. 1:**
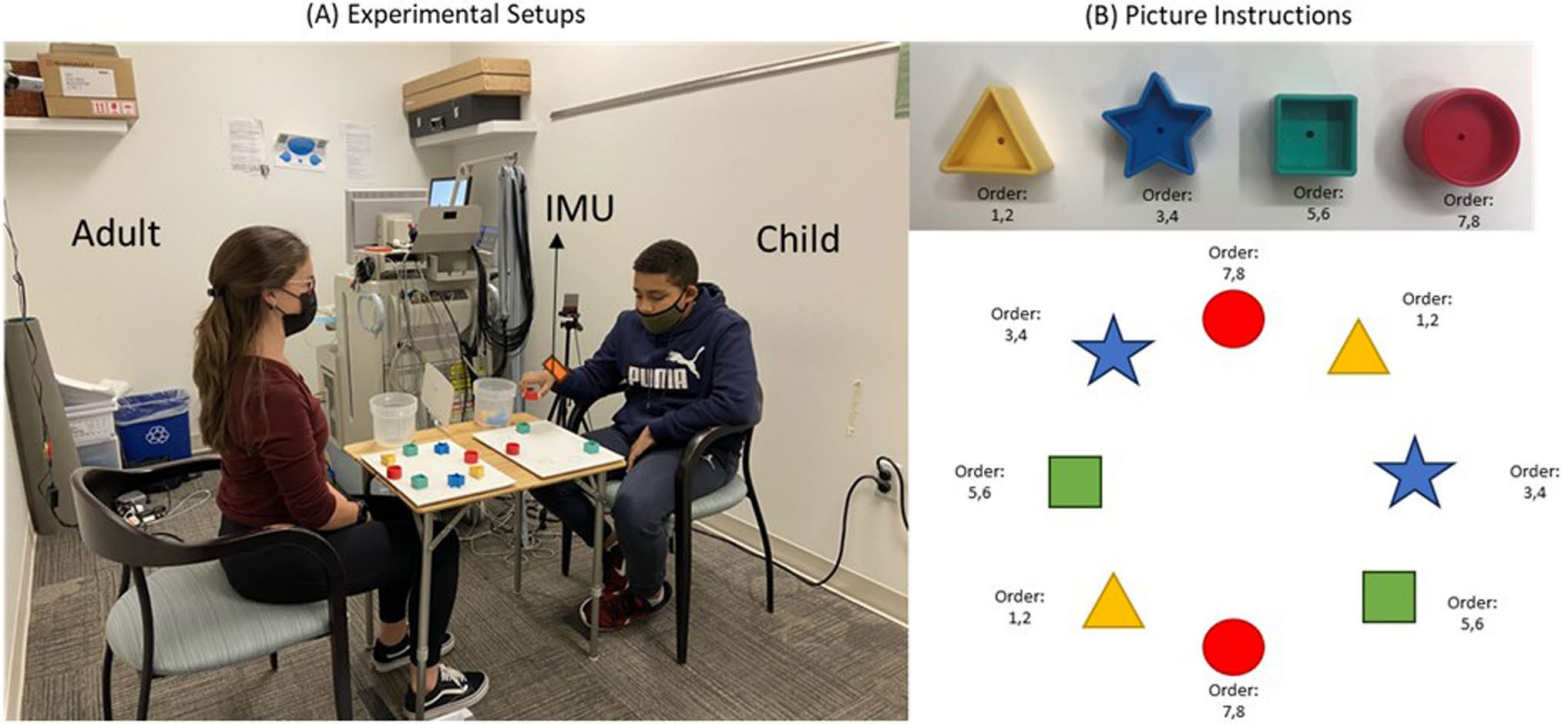
Experimental setups and examples for picture instruction.

Upon confirming eligibility, the participating children were seated at a table across from an adult experimenter. Both the child and adult reached for their own sets of blocks that were arranged in a circular manner, with the containers placed at the child’s right and on the adult’s left-hand side (as depicted in Fig. 1A). The child was instructed to use their right hand throughout the experiment, while the adults moved their left hand in order to mirror the child’s actions. The inertial measurement units (IMUs, Xsens, Inc.; sampling rate: 100 Hz) were fitted to the wrists of both the child’s and the adult’s moving hands (i.e., the child’s right and the adult’s left hand). During the experiment, the adult experimenter and the child took turns reaching for the blocks and placing them into the container one by one, according to pictorial instructions (Fig. 1B). E-prime software was used to trigger the start of each trial, and an experimenter marked the end of each trial when the child and experimenter finished putting all blocks into the containers. All children and adults completed a total of 6 reaching-placing trials. Note that any one out of 4 adult experimenters were randomly paired with the child participants during the reach-place task. The current study utilized a subset of data from our previous research, which investigated cortical activation and movement kinematics in children with ASD during action observation, execution, and imitation^37,38^. While this study only focuses solely on the kinematics of goal-directed, reach-place actions based on pictorial instructions that explain the sequence of blocks to be picked up (i.e., the execution condition), it is possible that the presence of an adult may influence children’s movement kinematics. Further research is needed to determine whether the presence of an adult affects children’s reaching and placing movements.

### Movement kinematics

The raw kinematic data includes wrist displacement in a 3D coordinate system, recorded by the IMU sensor. We first transformed the raw displacement data from each participant into a common reference coordinate system. Then, we computed twelve kinematic parameters from the IMU data collected during the reaching and placing tasks performed by both children and adults. Specifically, we calculated reaction time as the time interval between the initiation signal and the onset of movement. Movement initiation was determined when both the relative distance (in relation to the starting point) and acceleration spiked to 20% of their maximum values. Once movement initiations were identified, we computed additional statistics, including total distance, average velocity, maximum velocity, time to peak velocity, average acceleration, maximum acceleration, and time to peak acceleration. Furthermore, we assessed the number of movement units along the three motion vectors (X, Y, and Z). An individual movement unit was defined as the moment when a kinematic movement vector crosses zero, representing a significant change in direction. We calculated three types of movement units (Type 1 to Type 3), by detecting instances where velocity (Type 1), acceleration (Type 2), and jerk (Type 3) crossed zero in the x, y, and z vectors. To consolidate the zero-crossing incidence across these three axes, we calculated the Root Mean Squares (RMS) for each of the three movement unit types.

### Statistical analyses

We used Levene’s test and the Kolmogorov–Smirnov test to assess homogeneity and normal distribution of our data. Most of the variables did not pass the homogeneity test (Supplementary Table S1) and did not satisfy the assumptions of normal distribution (Supplementary table S2). Therefore, we used the non-parametric, Mann–Whitney U test for the between-group comparisons (Adult vs. TD; TD vs. ASD). In order to explore the associations between movement kinematics variables and the adaptive functioning of the children, Pearson correlations were conducted. All statistical analyses were conducted using IBM SPSS (Version 29, SPSS, Inc.; URL: http://www.spss.com).

### Deep learning model

Due to the limited number of training data, instead of using an end-to-end deep learning model to predict the ASD classification with the raw kinematic data, we used kinematic parameters as the input to the model. Nine out of the twelve kinematic parameters exhibited significant (or borderline significant) differences between children with and without ASD, as detailed in Result 3.1 and visualized in Fig. 3. These nine parameters were computed for all trials of reaching and placing tasks performed by the children, resulting in a dataset of approximately 221 samples. Approximately 65% of the dataset represented children with ASD, with the remaining portion comprising typically developing children. Prior to training, each statistic was standardized across the entire dataset using min–max normalization. This normalized dataset served as the foundation for training a straightforward Multilayer Perceptron (MLP) model. The model consisted of four fully connected layers, incorporating three batch normalizations interspersed among the layers, as well as three leaky ReLU activation functions. A sigmoid function was employed for the output layer. The ADAM optimizer was utilized, with the initial learning rate set at 1e–5, subsequently adjusting to 1e–6 once the training accuracy reached 95%. Each model underwent 200 epochs of training, and all accuracy evaluations were conducted through ten-fold cross-validation. To determine the significance of each feature, permutation feature importance was computed^39–41^. The model was fully trained for 200 epochs, and the lowest validation loss was recorded. Subsequently, each kinematic parameter was individually shuffled to introduce randomization, and the trained model was reevaluated for classifying the validation set. The difference between the initial best loss and the new loss resulting from the permutation of a feature represented its permutation feature importance.

## Results

### Movement kinematics

**Fig. 2:**
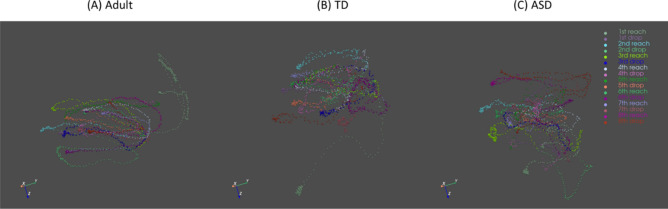
Movement trajectories of adults (**A**), typically developing children (**B**), and children with ASD (**C**).

**Fig. 3:**
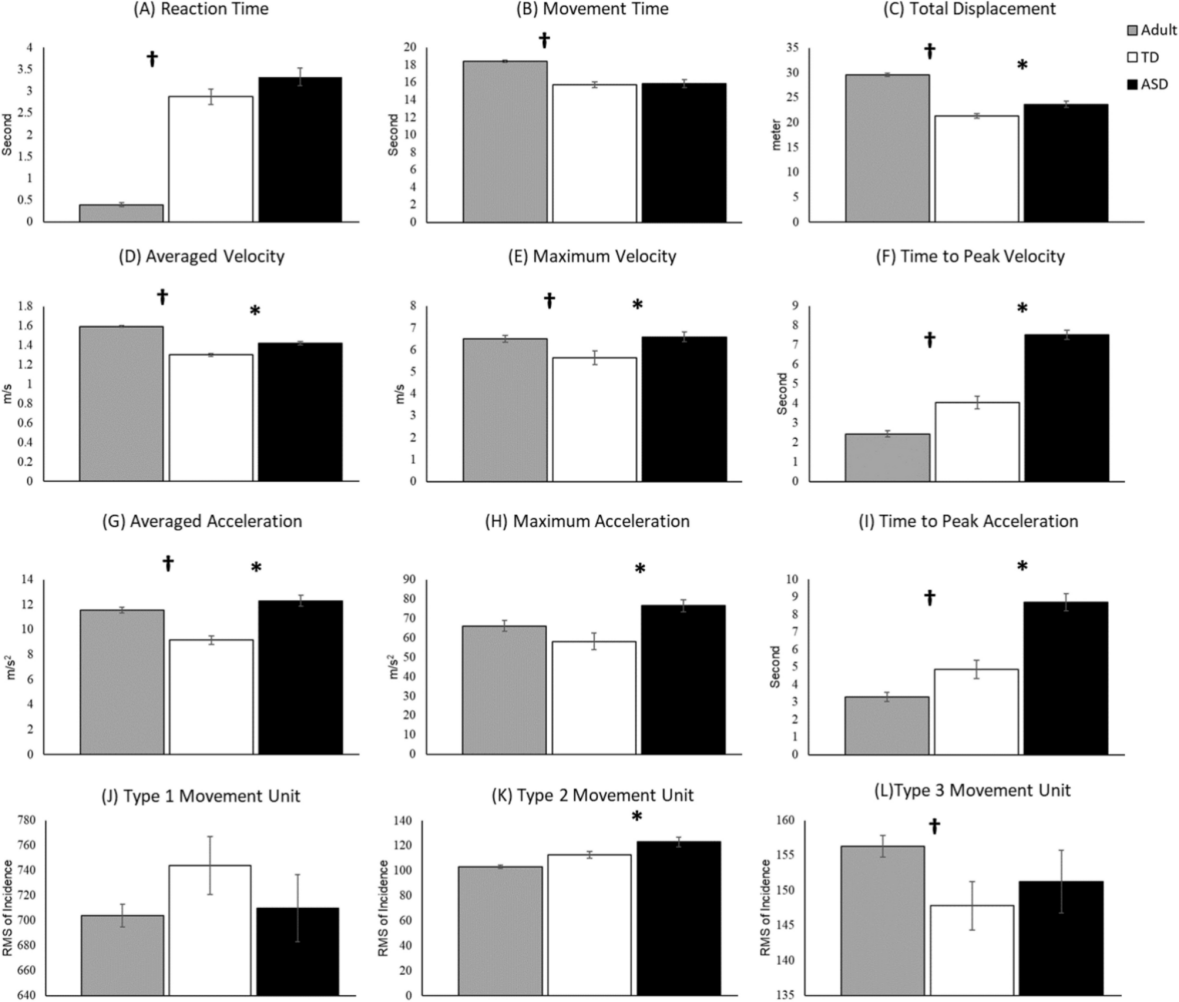
Movement kinematics for adult, TD children, and children with ASD, including reaction time (**A**), movement time (**B**), total displacement (**C**), averaged velocity (**D**), maximum velocity (**E**), time to peak velocity (**F**), averaged acceleration (**G**), maximum acceleration (**H**), time to peak acceleration (**I**), type 1 movement unit (**J**), type 2 movement unit (**K**), type 3 movement unit (**L**).

In Fig. 2, we presented the movement trajectories of adults, TD children, and children with ASD. Notably, children with ASD have wider and jerkier movement trajectories compared to both TD adults and children. Statistical analyses through a Mann–Whitney U test revealed significantly longer movement time, greater total displacement, averaged velocity, maximum velocity, and averaged acceleration, and type 3 movement unit in adults compared to the TD children (*p*s < 0.05; Fig. 3B–D, E, G, L). On the contrary, adults showed faster reaction time, time to peak velocity, time to peak acceleration compared to the TD children (*p*s < 0.05; Fig. 3A, F, I), suggesting developmental differences in temporal motor control parameters. For the ASD-related differences, children with ASD demonstrated increased values in nearly all kinematic parameters, including total displacement, averaged velocity, maximum velocity, time to peak velocity, averaged acceleration, maximum acceleration, time to peak acceleration, and Type 2 movement units (*p*s < 0.05; Fig. 3C–F, G–I, K). Additionally, a notable trend toward significance was observed in reaction time, suggesting that children with ASD required more time to initiate their movements (*p* = 0.09; Fig. 3A).

### Correlations between motor performance and adaptive functions

TD children showed significant correlations between the movement kinematics with the VABS Daily living and socialization scores, whereas children with ASD showed significant correlations between the VABC communication and socialization scores (*p*s < 0.001; Table 2). Specifically, in TD children, averaged velocity and acceleration were positively correlated with VABC social scores (r = 0.336 ~ 0.341, *p* < 0.001, Table 2), while the movement time, and movement units (Type 1 to 3) were negatively correlated with the VABS daily living and socialization scores (r = − 0.341 ~ − 0.525, *p* < 0.001; Table 2). For children with ASD, movement time, displacement, averaged velocity, averaged acceleration, and maximum acceleration were positively correlated with their VABS communication and/or socialization scores (r = 0.237 ~ 0.452, *p* < 0.001; Table 2).

**Table 2:** Correlations between the movement kinematics and children’s adaptive functioning.

	TD			ASD		
	VABS-Com	VABS-DL	VABS-Soc	VABS-Com	VABS-DL	VABS-Soc
Reaction time	− 0.229	0.014	0.096	0.028	− 0.011	− 0.127
Movement time	− 0.293*	− 0.525**	− 0.482**	0.178*	0.056	0.242**
Displacement	− 0.194	− 0.273*	− 0.166	0.324**	0.135	0.452**
Ave velocity	0.159	0.289*	0.336**	0.256**	0.137	0.398**
Max velocity	0.007	− 0.022	0.118	0.100	0.008	0.176*
Time to peak velocity	0.133	0.092	− 0.079	0.060	0.017	0.099
Ave acceleration	0.247*	0.268*	0.341**	0.197*	0.094	0.372**
Max acceleration	0.124	− 0.010	0.171	0.152	0.029	0.237**
Time to peak acceleration	0.111	0.018	0.028	0.086	− 0.039	0.139
Type 1 movement unit	− 0.283*	− 0.525**	− 0.460**	0.012	− 0.066	0.040
Type 2 movement unit	− 0.147	− 0.421**	− 0.321**	0.065	− 0.048	0.190*
Type 3 movement unit	− 0.224	− 0.491**	− 0.438**	0.134	0.019	0.212*

*indicates *p*-value < 0.05; **indicates *p*-value < 0.001; Bold value indicates *p*-value survived FDR correction.

### Classification of adults and children

**Fig. 4:**
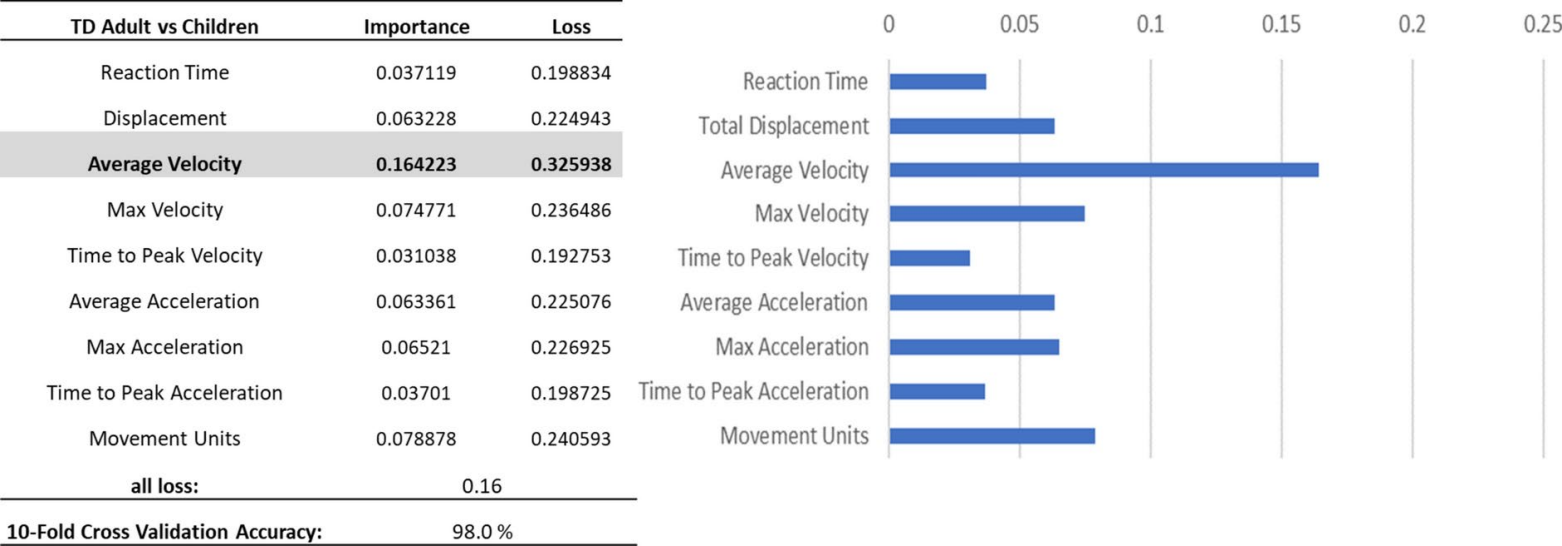
Feature importance analyses for classifying adults vs TD children.

The deep learning model achieved a tenfold cross-validation accuracy of 98.00% in classifying between adults and TD children and 98.62% in classifying adults and children with ASD. Feature importance analyses showed that the averaged velocity is the most important factor for the developmental classification (Fig. 4; Supplementary Figure S2).

### Classification of children with and without ASD

**Fig. 5:**
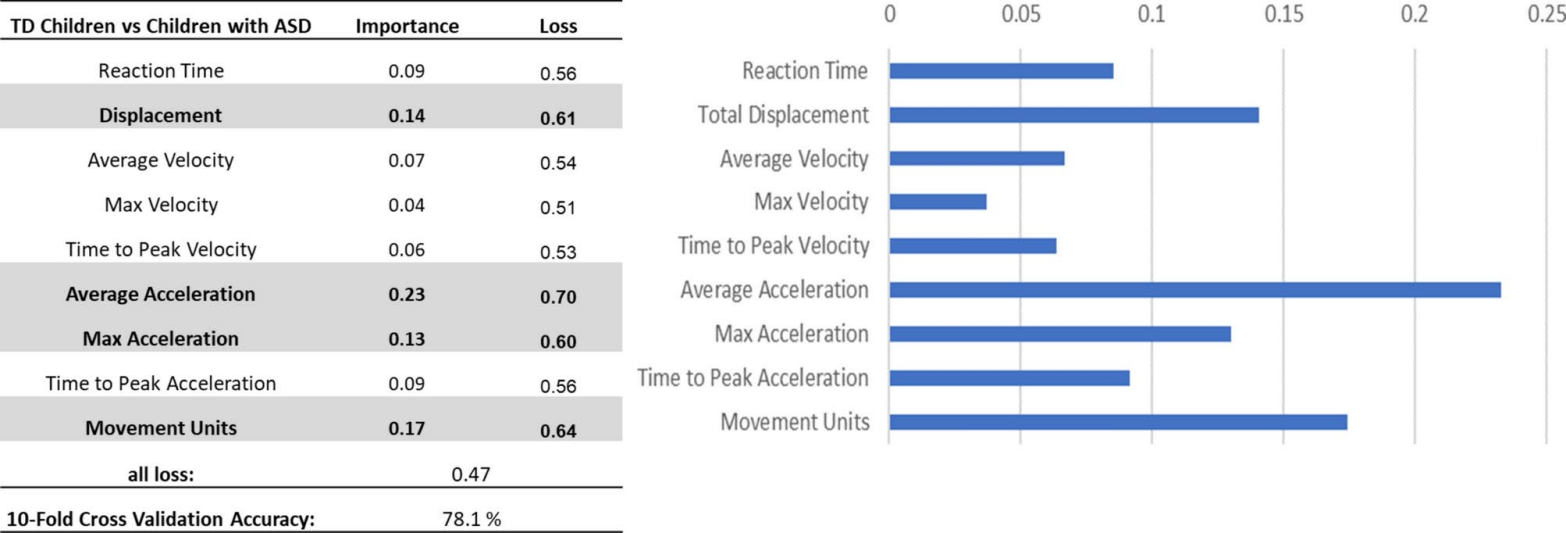
Feature importance analyses for classifying TD children vs children with ASD.

The deep learning model achieved a tenfold cross-validation accuracy of 78.1% in classifying between children with and without ASD. Feature importance analyses showed that the Averaged Acceleration, followed by Movement Units, Displacement, and Maximum Acceleration are the important factors for the TD vs ASD classification (Fig. 5).

## Discussion

Autism Spectrum Disorder (ASD) is one of the most prevalent neurodevelopmental disorders, necessitating objective diagnostic approaches. By integrating reaching kinematic measures with deep learning methods, this study aimed to explore the relationship between adaptive functioning and motor kinematics in children with and without ASD, providing action-based, objective biomarkers of ASD. Our findings showed many differences in reaching kinematics of school-aged children with ASD compared to TD children/adults, with children having longer reaction times and time to peak velocity/acceleration, shorter movement times and total displacements, lower average velocity/acceleration and maximum acceleration, as well as fewer movement units compared to healthy adults. When comparing children with and without ASD, children with ASD showed greater total displacement, averaged velocity/acceleration, maximum velocity/acceleration, time to peak velocity/acceleration, as well as movement units compared to the TD children. Reaching kinematics also correlated to the daily living and socialization scores of TD children and to the communication and socialization scores of children with ASD. More importantly, our deep learning models achieved ~ 98% accuracy in classifying TD children and adults and ~ 78.1% in classifying children with and without ASD. Our findings support the use of reaching kinematics and deep learning methods in classifying children with and without ASD and have the potential to assist in diagnosis/early identification process upon further validation of their specificity among younger children.

### Developmental differences in goal-directed reaching movements

In line with prior research^15,42,43^, the current study highlights the ongoing refinement of upper limb movements in school age children. In fact, greater classification accuracy was observed between adults and children (both with and without ASD) compared to the classification between children with and without ASD, suggesting that developmental differences in motor planning and control during goal-directed movements are more pronounced than ASD-related differences. Specifically, we observed a longer reaction time in TD children compared to healthy adults, indicating delayed anticipatory control of reaching. Anticipatory control of movements also involves motor planning /executive functioning processes such as motor planning, inhibitory control, working memory, and cognitive shifting, that are known to stabilize in early adulthood^44^. Therefore, consistent with other studies, slower reaching reaction times in children relative to adults^45^, aligns with the expected developmental trajectory of anticipatory control and executive functioning. We also found shorter movement time and total displacement in children compared to adults, findings that are counterintuitive and not consistent with previous findings^46^. This discrepancy may stem from the unique experimental setup of the study, wherein adult participants were paired with children to complete the reaching and placing task. It is conceivable that the adults exaggerated their movements for the benefit of the child partner, thus executing movements with larger amplitude and taking more time to complete the task. Despite the shorter movement times in children, they moved slower and jerkier and showed more type 1 and type 2 movement units compared to healthy adults, a characteristic consistently observed in previous studies, suggesting insufficient online movement control^15,47^. Moreover, children demonstrated distinct temporal parameters compared to adults during the reaching and placing task, featuring lower averaged velocity/acceleration, maximum velocity, and longer time to peak velocity/acceleration. These findings align with previous kinematic observations, indicating insufficient feedforward/anticipatory estimates of movement trajectories in the service of online movement control^42,43^.

### ASD-related differences in goal-directed reaching movements

Compared to TD children, children with ASD differed in their kinematics during the reach-place task as seen by the prolonged reaction times, increased time to peak velocity/acceleration, and higher frequency of movement units. Delayed executive functioning/motor planning in children with ASD could also contribute to the longer reaction times, as highlighted in studies by Demetriou et al. and Willoughby et al.^48,49^. Recent findings indicate children with ASD have more difficulties with anticipatory movement control compared to TD children. Challenges in utilizing visual information for anticipatory/feedforward control of movements are evident in children with ASD may have led to extended time to peak velocity/acceleration and more substantial online movement corrections^23,25^. For instance, during interceptive tasks, children with ASD did not rely on visual cues for movement planning and had greater movement units, as was observed in the study by Chen et al.^25^. Children with ASD demonstrated a unique movement pattern of increased movement speed/acceleration (i.e., greater averaged and maximum velocity/acceleration), and greater movement overshooting (i.e., greater total displacement) compared to their TD peers. These findings, consistent with previous studies using horizontal sinusoidal arm movements and interceptive tasks^25,50^, may signify an unique strategy employed by children with ASD to overcome the delays in movement initiation. In summary, children with ASD’s delays in movement initiation, coupled with faster yet less fluid movements, may result in overshooting as well as lower accuracy.

### Relationships between movement kinematics and adaptive functioning

Consistent with past findings^5,6^, our study found significant correlations between children’s motor skills and adaptive functioning. Specifically, TD children with higher VABS social scores demonstrated greater averaged velocity and acceleration, shorter movement times, and fewer movement units during reach-and-place movements. In contrast, children with ASD who had higher VABS communication and/or socialization scores showed longer movement times, displacement, averaged velocity and acceleration, and maximum acceleration during reach-and place-movements. These connections highlight the fundamental role of motor skills in overall functioning as well as its inter-dependence with other developmental domains^27^. For example, the acquisition of new motor skills, such as manual dexterity and locomotor skills, not only fosters exploration and learning but also exerts a cascading influence on social communication, cognitive abilities, and overall functioning^26^. While a general correlation exists between motor skills and adaptive functioning in both TD and ASD groups, the nature of these relationships differed for children with and without ASD. Specifically, TD children exhibiting better adaptive functioning and socialization skills displayed more advanced movement kinematics such as shorter movement times, greater velocity/acceleration, and reduced movement units. In contrast, among children with ASD, those with better social communication performance exhibited prolonged movement times, greater displacement, velocity, and acceleration. This suggests that children with higher functioning ASD may employ unique mechanisms during the execution of the reach-place task.

### Deep learning classification and future implications

Despite the growing body of literature on motor difficulties in children with ASD, the translation of these findings to effective tools for early identification remains limited. One challenge is ensuring the specificity of these motor difficulties in children with ASD, distinguishing them from those seen in other developmental disorders. Some researchers have advocated for the inclusion of motor difficulties as diagnostic criteria or specifiers; however, the evaluation of motor difficulties still heavily relies on parent reports and subjective behavioral assessments^5–7^. To our knowledge, this is the first study to leverage continuous upper limb movement kinematics and deep learning methods for the classification of individuals with and without ASD. Our findings underscore the feasibility of employing a comprehensive examination of movement kinematics and deep learning methods to classify children with ASD which might facilitate ASD diagnosis upon validation in younger children.

While the current study included a reasonable sample size for an investigation centered on movement kinematics (N = 41), the group sizes were uneven and may be considered relatively small for training an end-to-end deep learning model or using other network designs for time-series data such as Long Short-Term Memory network (LSTM). Future research endeavors should include larger and more balanced sample sizes across groups to enhance the optimization of deep learning models. Moreover, although we used VABS-2 to assess children’s adaptive functioning, we did not include specific measures of intellectual ability. Future studies should incorporate IQ to further explore how intellectual ability may influence goal-directed movements in children. Beyond dataset expansion, several improvements could be made to the deep learning model. The current model requires pre-processing and identification of important kinematic parameters before putting them into the model. While this approach is beneficial for determining differences in movement strategies among adults, TD children, and children with ASD, it may also limit the maximum accuracy of the model. Future deep learning models could be developed to train on the raw kinematic data, allowing the model to autonomously calculate internal parameters optimized for classification. For example, an ideal architecture for such a model might incorporate recurrent layers, configured into an autoencoder with an attention mechanism to mitigate the risk of vanishing gradients. Lastly, to apply these methods for early identification of ASD in clinical settings, their specificity in diagnosing ASD—distinct from other developmental disorders like developmental coordination disorder—must be verified. Future studies that include children with ASD and other motor difficulties, such as developmental coordination disorder, could further confirm the specificity of these methods in screening for ASD. These refinements stand as potential avenues for advancing the efficacy and efficiency of using kinematic parameters and deep learning models in classifying children with and without ASD and facilitating ASD diagnosis upon validation in younger children.

## Conclusions

Using a continuous reach and place paradigm, the current study found differences in movement kinematics among TD children compared to healthy adults, revealing the ongoing refinement of anticipatory control and motor planning throughout school age. More importantly, children with ASD exhibited different profiles indicating less refined anticipatory control and motor planning, as seen by movement overshooting, prolonged time to peak velocity/acceleration, and a higher frequency of movement units. Interestingly, children with ASD also demonstrated unique movement strategies, employing increased velocity and acceleration to offset their reaching difficulties. More importantly, the implementation of a deep learning model yielded a noteworthy 78% accuracy in effectively classifying children with ASD from TD children, thereby substantiating the potential utility of movement kinematics and deep learning as an objective screening tool for ASD diagnosis, upon further validation in younger children. These findings hold promise for improving early identification methods and underscore the significance of using objective measures to quantify motor difficulties in children with ASD.

## Supplementary Information

Supplementary Information.

## Data Availability

The datasets generated during and/or analyzed during the current study are available from the corresponding author on reasonable request.
