# Supplementary material for: Using deep learning to classify developmental differences in reaching and placing movements in children with and without autism spectrum disorder
Source: Sci Rep. 2024 Dec 5;14:30283. doi: 10.1038/s41598-024-81652-z (PMC11618337; doi:10.1038/s41598-024-81652-z)

**Developmental Differences in Reaching-and-Placing Movement and Its Use in Classifying Children with and without Autism Spectrum Disorder: Deep Learning Approach**

**Wan-Chun Su,^1,2^ John Mutersbaugh,^1^ Wei-Lun Huang^1,3^, Anjana Bhat^4,5,6^, Amir Gandjbakhche ^1^**

**Supplementary Table S1.** Levene Test for Homogeneity

|  | Levene Test | | |
| --- | --- | --- | --- |
|  | Statistic | df1/df2 | p-value |
| Reaction Time | 56.865 | 2/440 | <0.001* |
| Movement Time | 51.557 | 2/440 | <0.001* |
| Total Displacement | 21.578 | 2/440 | <0.001* |
| Averaged Velocity | 14.950 | 2/440 | <0.001* |
| Maximum Velocity | 4.385 | 2/440 | <0.013* |
| Time to Peak Velocity | 31.659 | 2/440 | <0.001* |
| Averaged Acceleration | 22.030 | 2/440 | <0.001* |
| Maximum Acceleration | 1.519 | 2/440 | 0.220 |
| Time to Peak Acceleration | 15.759 | 2/440 | <0.001* |
| Type 1 Movement Unit | 47.297 | 2/440 | <0.001* |
| Type 2 Movement Unit | 37.154 | 2/440 | <0.001* |
| Type 3 Movement Unit | 49.309 | 2/440 | <0.001* |

**Supplementary Table S2.** Kolmogorov-Smirnov Normal Distribution Test

|  | Adult | | | TD | | | ASD | | |
| --- | --- | --- | --- | --- | --- | --- | --- | --- | --- |
|  | Statistic | df | p-value | Statistic | df | p-value | Statistic | df | p-value |
| Reaction Time | 0.278 | 225 | <0.001* | 0.103 | 219 | <0.001* | 0.125 | 144 | <0.001* |
| Movement Time | 0.074 | 224 | 0.005* | 0.091 | 219 | <0.001* | 0.106 | 144 | <0.001* |
| Total Displacement | 0.096 | 224 | <0.001* | 0.147 | 2019 | <0.001* | 0.144 | 144 | <0.001* |
| Averaged Velocity | 0.082 | 224 | <0.001* | 0.060 | 219 | 0.051 | 0.068 | 144 | 0.200 |
| Maximum Velocity | 0.174 | 224 | <0.001* | 0.171 | 219 | <0.001* | 0.133 | 144 | <0.001* |
| Time to Peak Velocity | 0.316 | 224 | <0.001* | 0.145 | 219 | <0.001* | 0.110 | 144 | <0.001* |
| Averaged Acceleration | 0.147 | 224 | <0.001* | 0.091 | 219 | <0.001* | 0.084 | 144 | 0.014* |
| Maximum Acceleration | 0.199 | 224 | <0.001* | 0.135 | 219 | <0.001* | 0.117 | 144 | <0.001* |
| Time to Peak Acceleration | 0.311 | 224 | <0.001* | 0.115 | 219 | <0.001* | 0.102 | 144 | <0.001* |
| Type 1 Movement Unit | 0.112 | 224 | <0.001* | 0.105 | 219 | <0.001* | 0.119 | 144 | <0.001* |
| Type 2 Movement Unit | 0.208 | 224 | <0.001* | 0.148 | 219 | <0.001* | 0.131 | 144 | <0.001* |
| Type 3 Movement Unit | 0.095 | 224 | <0.001* | 0.109 | 219 | <0.001* | 0.106 | 144 | <0.001* |

*Indicates *p* value < 0.05.

**Supplementary Table S3.** Mann-Whitney U Test for Between Group (Adult vs. TD & TD vs. ASD) Comparisons

|  | Adult vs. TD | | TD vs. ASD | |
| --- | --- | --- | --- | --- |
|  | U Statistics | p-value | U Statistics | p-value |
| Reaction Time | 1576.0 | <0.001* | 4922.5 | 0.283 |
| Movement Time | 3611.5 | <0.001* | 5053.5 | 0.436 |
| Total Displacement | 1255.0 | <0.001* | 4579.0 | 0.065+ |
| Averaged Velocity | 1323.0 | <0.001* | 3485.0 | <0.001* |
| Maximum Velocity | 5059.0 | <0.001* | 3858.0 | <0.001* |
| Time to Peak Velocity | 6158.5 | <0.001* | 3307.5 | <0.001* |
| Averaged Acceleration | 4620.0 | <0.001* | 3225.0 | <0.001* |
| Maximum Acceleration | 6728.0 | 0.010* | 3332.0 | <0.001* |
| Time to Peak Acceleration | 6139.0 | <0.001* | 3177.0 | <0.001* |
| Type 1 Movement Unit | 7875.5 | 0.418 | 4837.5 | 0.206 |
| Type 2 Movement Unit | 7704.5 | 0.283 | 4348.0 | 0.018* |
| Type 3 Movement Unit | 5842.0 | <0.001* | 5330.5 | 0.876 |

*indicates significance (*p* < 0.05), † indicated borderline significance (0.05 < *p* < 0.1).

**Supplementary Figure S1.** Correlations Between the Movement Kinematics and the Adaptive Functioning in Children with ASD.

**
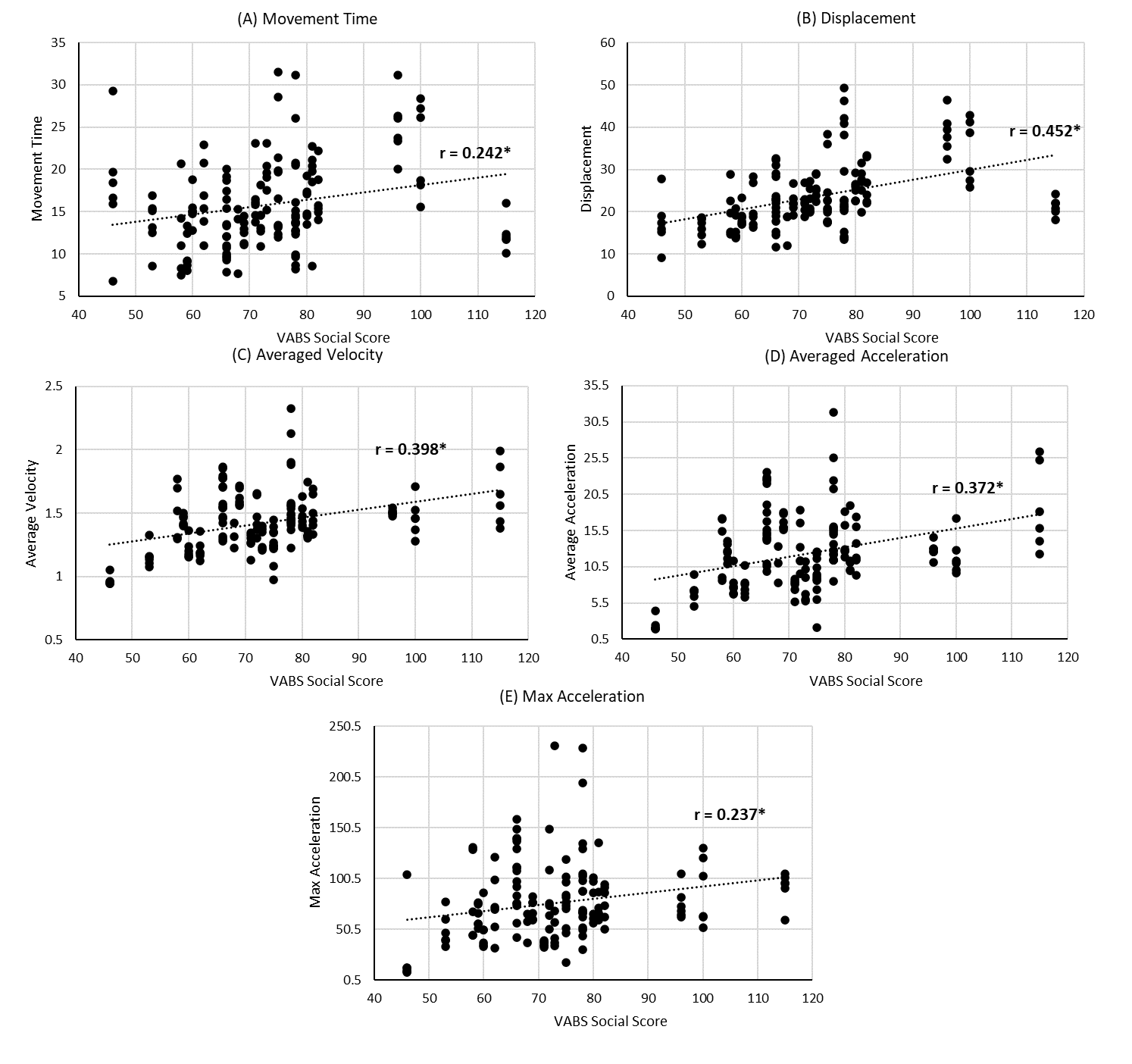
**

**Supplementary Figure S2.** Feature importance analyses for classifying adults vs children with ASD


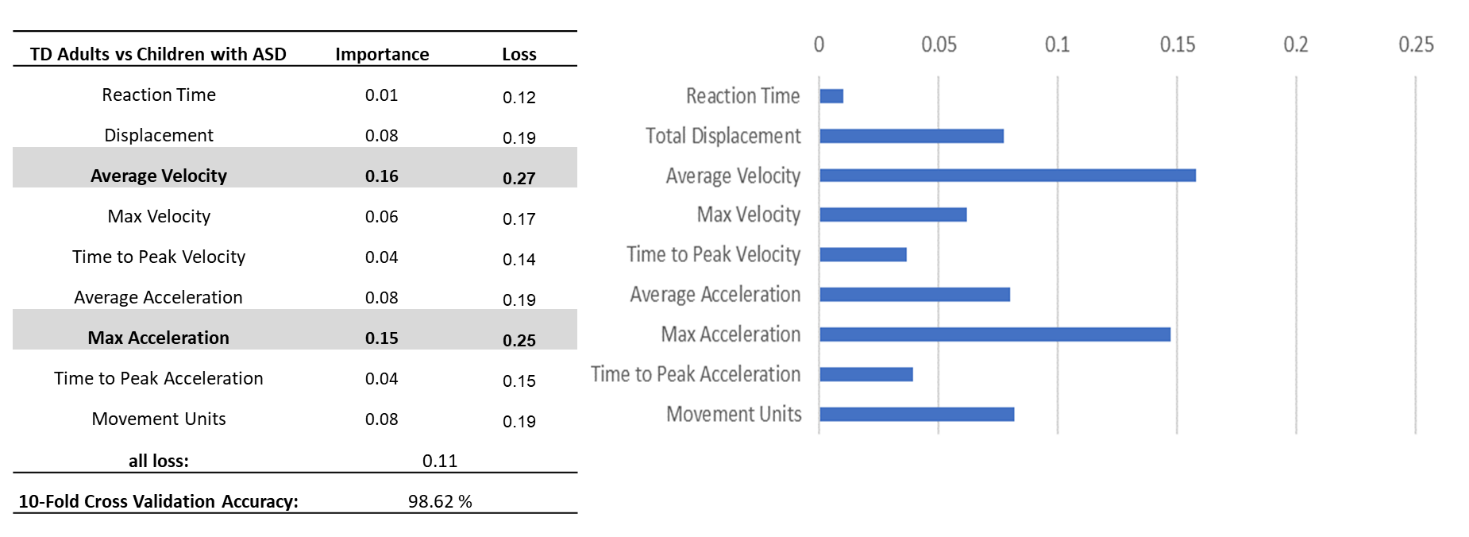

Supplement: Supplementary file 1 — Supplementary Information. [file 41598_2024_81652_MOESM1_ESM.docx]
